# Individual, community and service environment factors associated with modern contraceptive use in five Sub-Saharan African countries: A multilevel, multinomial analysis using geographically linked data from PMA2020

**DOI:** 10.1371/journal.pone.0218157

**Published:** 2019-06-20

**Authors:** Linnea A. Zimmerman, Suzanne O. Bell, Qingfeng Li, Antonia Morzenti, Philip Anglewicz, PMA2020 Principal Investigators Group, Amy O. Tsui

**Affiliations:** 1 Department of Population, Family and Reproductive Health, Johns Hopkins Bloomberg School of Public Health, Baltimore, Maryland, United States of America; 2 Department of International Health, Johns Hopkins Bloomberg School of Public Health, Baltimore, Maryland, United States of America; 3 Center for Communication Programs, Department of Health, Behavior, and Society, Johns Hopkins Bloomberg School of Public Health, Baltimore, Maryland, United States of America; University of Cape Coast, GHANA

## Abstract

The importance of the family planning service environment and community-level factors on contraceptive use has long been studied. Few studies, however, have been able to link individual and health facility data from surveys that are nationally representative, concurrently fielded, and geographically linked. Data from Performance Monitoring and Accountability 2020 address these limitations. To assess the relative influences of the service delivery environment and community, household, and individual factors on a woman’s likelihood of using a modern contraceptive in five geographically and culturally diverse sub-Saharan African countries. Nationally representative, cross-sectional data from PMA2020 were linked at the household and service delivery level. Country-specific and pooled multilevel multinomial logistic models, comparing non-users, short- and long-acting method users were used. The variables elected for inclusion in our multivariate analyses were guided by the conceptual framework to profile the different levels of influences on individual use of modern contraception. Average marginal effects were calculated to improve interpretability. We find that the effect of contextual factors varies widely but that being visited by a health worker who spoke about family planning in the past 12 months was consistently and positively associated with individual use of short-acting and long-acting contraception. Characteristics of the nearest health facility did not generally exercise their own independent influences on a woman’s use of contraception, except in the case of Burkina Faso, where the average distance between individuals and the nearest family planning provider was significantly greater than other countries. Inclusion of country fixed effects in the pooled models and the relevance of covariates at different levels in the country-specific models demonstrate that there is significant variation across countries in how community, individual, and service delivery environment factors influence contraceptive use and method choice. Context must be taken into account when designing family planning programs.

## Introduction

The importance of the family planning service environment and community-level factors on fertility and contraceptive use for low- and middle-income countries (LMICs) has long been studied [1–5]. This body of research has highlighted the influence of service environment factors (e.g. proximity, quality, and facility type), on a range of female contraceptive outcomes, including current practice, choice of method, and duration of use. Community-level measures of socioeconomic development (e.g. mean education level, mean household wealth, and religious composition) and contextual factors (e.g. gender norms, spouse communication, and social capital) have been found to be associated with modern method use [6] and consistent use [7]. The knowledge base that has accumulated over the past decades has grown in rigor with the application of advanced statistical methods for model estimation, in particular hierarchical linear modeling or multi-level analysis (MLA) to address the nested data structure of individuals within households and households within communities. MLA has enabled the assessment of cross-level influences of higher-level observed factors on lower level individual outcomes and of unobserved variation at multiple levels. In addition, improvements in computational efficiency have facilitated the estimation of multilevel factors’ influences after adjusting for complex survey design.

Much of the multilevel research on the utilization of reproductive and maternal health services, such as modern contraception, facility-based delivery, and births attended by skilled health providers, has relied on data collected through the Demographic and Health Surveys (DHS) using both the household and female surveys as well as the Service Provision Assessment (SPA) [8–11]. DHS data offer several important benefits, such as nationally-representative samples, standardized measures across country surveys and over time, and coverage of a broad range of health issues. Community-level measures are often based on data for the sample enumeration areas or clusters, wherein these are assumed to represent the “community” of the survey respondent or household. Measures are constructed by aggregating and summarizing data for a given cluster using the sampled individuals or households, such as mean number of years of female schooling or proportion of sampled households in the lowest wealth quintile. The DHS SPA survey contains a wealth of information on health facilities sampled from subnational areas, such as districts, and has generally been conducted with an independent sample design and at a different time from the DHS household survey. Occasionally the SPA will be a census of facilities, as in Haiti in 2013 [12].

Despite the useful possibilities for multilevel data analysis using DHS or SPA data, there are important limitations to these datasets. Multilevel analyses that incorporate both the service environment and individual and community factors rely on the ability to link health facility information from the SPA to the individual or household record from the DHS. While there are various procedures for linking data, the availability of geo-location data for both the survey cluster and the health facilities improves the accuracy in profiling the service environment of the household and individual [8]. Analysis of linked data can then address important supply-demand questions, such as which facility, service, or provider characteristics influence health care utilization and result in improved health outcomes. These types of programmatic findings are needed to inform the planning and efficient and effective use of scarce resources. The ability to effectively link this information using the DHS alone however is limited. Household and facility surveys are often fielded several years apart and linkages may involve too long an interval to support a temporally cogent analysis. Additionally, the selection of households and facilities are done separately, meaning that geographic coverage between the two surveys may be different or the SPA facility sample may be geographically dispersed and not spatially relevant to households in the DHS clusters. Finally, for reasons of confidentiality, GPS coordinates from the household survey in the DHS are only available at the cluster level, limiting the ability to analyze and account for individual variation in the service delivery environment.

Data from Performance Monitoring and Accountability 2020 address many of the limitations of reproductive health household surveys [13]. PMA2020 is a multi-country survey program that annually monitors key family planning indicators related to individual contraceptive use and intentions to practice and client service experiences. It also monitors facility level indicators related to service preparedness, such as the range of services provided, contraceptive supplies in stock, and integration of family planning with other primary sexual, reproductive, and maternal health services. Households are selected from randomly identified enumeration areas and both public and private facilities that serve the enumeration area are included in the sample. The two surveys are fielded concurrently and linking the two records offers an opportunity to pursue an in-depth investigation of the relationship between family planning services provided at proximal facilities with individual practice.

PMA2020 monitoring has shown recent increases in contraceptive use in a number of sub-Saharan African countries [14]. These increases do not appear to be due to survey or measurement error. Measures such as current use of any or a modern method comport well with DHS results generated for common survey years (see www.pma2020.org/fp-briefs). Such changes in both the levels of contraceptive practice and the composition of methods adopted in countries, e.g., Ethiopia, Uganda, Kenya, Burkina Faso, and Niger, warrant in-depth examination into the roles that service factors and other contextual influences play in facilitating use. Given longstanding resource constraints, the weakness of health systems in the region and low priority accorded to family planning investments by governments in the past, understanding how programmatic, as compared to community and individual, factors may have influenced a woman’s adoption of modern contraception becomes particularly intriguing. PMA2020 linked household and facility data lend themselves to such an investigation since they include not only significant detail on quality of service provision but also measured distances from households to health facilities, as well as female fertility preferences and contraceptive method concerns.

### Study aim

The aim of this study is to assess the relative influences of contextual factors, specifically the service delivery environment and community factors, household, and individual factors on a woman’s likelihood of using a modern contraceptive in five geographically and culturally diverse sub-Saharan African countries. A second aim is to assess whether these same factors operate differently for method choice as for use; that is, how do these factors influence the choice of short- or long-acting method. We expand upon previous research by using recent nationally representative household, female, and health facility survey data from these countries observed to have changing patterns of contraceptive practice.

## Methods

### Conceptual framework

**Fig 1:**
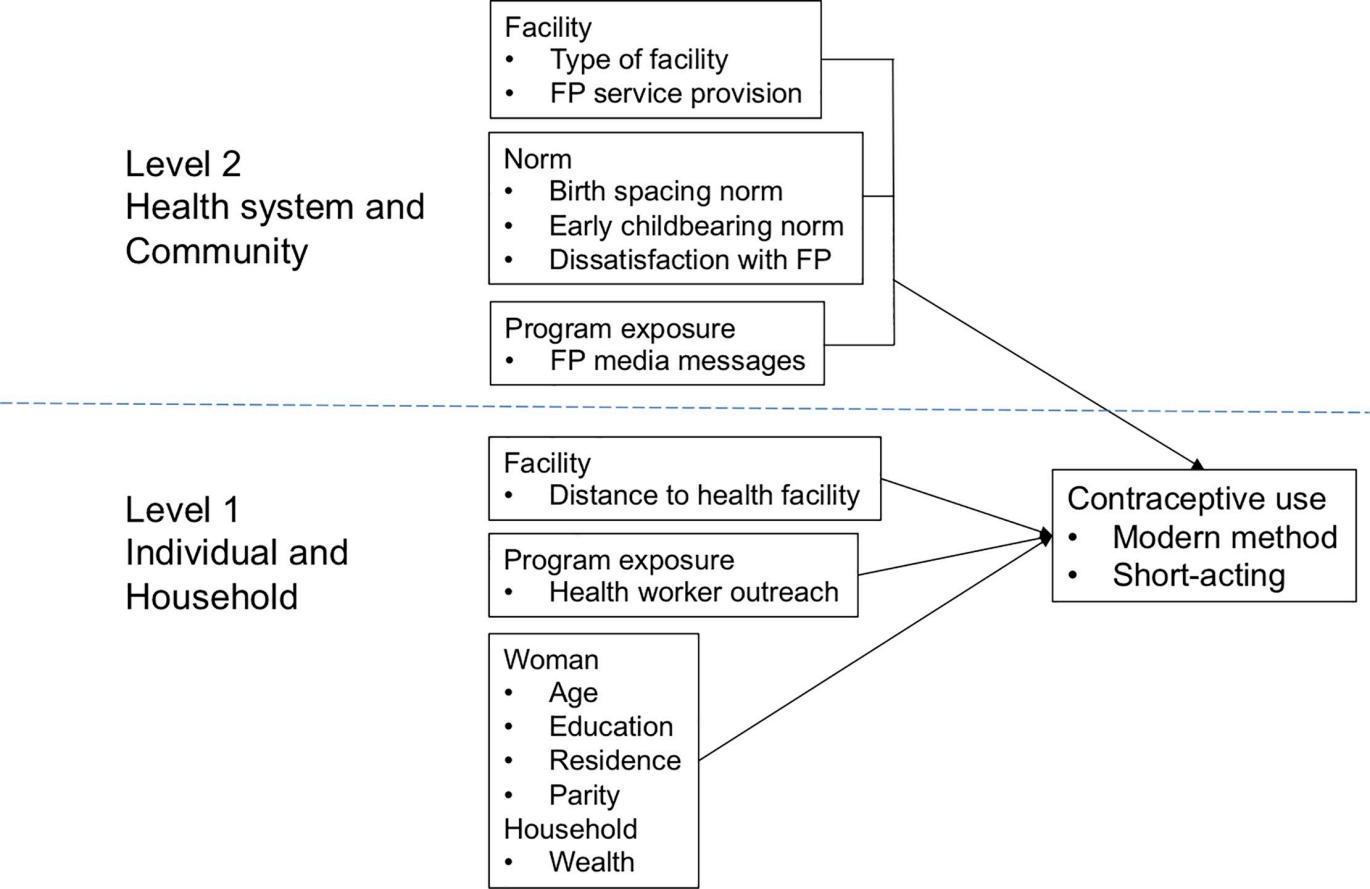
Multi-level framework of factors influencing individual contraceptive practice. Reprinted from Paek et al under a CC BY license, with permission from Paek, original copyright 2008.

Three sets of factors are conceptualized as influencing individual decisions to use contraception (Fig 1). The first set measures individual socio-demographic and household characteristics. The second includes community factors representing reproductive and family planning norms. The third set captures program exposure measured at the individual level and health system characteristics measured at the service delivery point (SDP) level. Program exposure factors include individual exposures to demand-generating activities, such as media messages about family planning or recent contact with health workers about family planning, and to health facility characteristics and service provision measures. These factors are hypothesized to operate across the community and individual levels.

### Data

PMA2020 surveys use a multi-stage stratified cluster design to draw a probability sample of households and females of childbearing age. They constitute a large-scale, nationally representative survey platform that is currently fielded in eleven geographies across Asia and Africa. Female resident enumerators (REs) conduct semi-annual or annual surveys at the household and individual female level and at SDPs in or near the communities in which they live. The REs use smartphones to conduct the interviews, which enables capturing GPS points at each sampled household or SDP. Datasets are made publicly available within six months of data collection via www.pma2020.org. Five countries are included in this analysis,–Burkina Faso, Ethiopia, Ghana, Kenya, and Uganda (R4 2016). We used data from the third round of the service delivery point survey and the fourth round of the female survey, conducted six months apart, so that we could assess how the service delivery environment affected subsequent contraceptive use within a six-month time period. The data management team within PMA used GPS coordinates to generate the distance between each household and the nearest service delivery point, but the individual coordinates were not made available to those outside of the data management team. Only the de-identified data with the addition of the distance measure, without linkage to the GPS coordinates, were used in the analysis.

A sample of enumeration areas (EAs) in each country is drawn to provide representative estimates of the modern contraceptive prevalence rate (mCPR) of all women at the national, and in some cases, sub-national level. The sample was drawn by the national statistical agency or its equivalent in each of the five countries. Enumeration areas are approximately 200 households in size. Once an EA is selected, the REs map and list every household within the EA to create a household sampling frame from which 33–44 households are randomly selected. The RE completes a household roster for each household selected for interview, and all females between the ages of 15 and 49 who are either usual members of the household or who slept in the household the night before are consented for interview using an individual questionnaire. Only females who are usual members of the household and who slept in the household the night before were included in this analysis.

Three types, or levels, of public health facilities are included in the PMA sample; the lowest level health facility, generally a health post, the next lowest-level, generally equivalent to a primary health center, and the district hospital. Facilities are selected into the PMA sample if their catchment area includes the sampled EA. PMA2020 supervisors conduct the public SDP interviews. During the EA listing process, all private SDPs that fall within the geographic boundaries of the EA are also included, and up to three are randomly selected assigned to the RE to interview. Private SDPs are included into the sample if they offer either general health services and commodities or are specialized maternal and reproductive health services and generally include pharmacies. dispensaries, and private health clinics.

This study’s analytic samples are all females age 15 to 49 years who slept in the household the night before and all public and private SDPs from the previous survey round. Guests to the household are excluded as the contextual factors that influence their contraceptive use may be different than those measured where they were visiting at the time of the survey. Sample sizes from each country are included in Table 1 along with other individual, household and SDP sample characteristics.

**Table 1:** Sample composition by variable type and country: Females age 15–49.

		Burkina Faso		Ghana		Ethiopia		Uganda		Kenya	
		2016		2015		2015		2016		2015	
Variable		%	SE	%	SE	%	SE	%	SE	%	SE
**Individual demographic variables**		n =	3,114	n =	5,048	n =	7,328	n =	3,651	n =	4,831
Modern contraceptive use		22.0	0.8	23.4	1.3	26.4	1.2	27.3	1.3	45.9	1.4
Percent of method mix—short-acting^1^		51.5	2.6	77.5	2.1	73.2	1.8	75.5	1.9	64.7	1.7
Female age (mean years)		28.6	2.4	28.6	0.2	27.9	0.2	28.4	0.2	28.1	0.2
Highest schooling level											
	No schooling	64.5	—	18.6	—	41.9	—	9.1	—	3.9	—
	Primary	16.1	—	18.0	—	38.4	—	62.6	—	51.0	—
	Secondary or more	19.4	—	63.4	—	19.7	—	28.3	—	45.1	—
Parity											
	0	22.5	—	36.2	—	32.6	—	22.1	—	28.9	—
	1–2	25.0	—	31.8	—	23.6	—	27.1	—	34.4	—
	3–4	21.5	—	20.4	—	16.3	—	20.8	—	22.0	—
	5+	31.0	—	11.6	—	27.6	—	30.0	—	14.6	—
Household wealth category											
	Lowest	20.4	—	22.3	—	19.4	—	19.7	—	21.3	—
	Middle lowest	19.7	—	20.3	—	19.3	—	19.0	—	21.1	—
	Middle	20.2	—	19.8	—	19.2	—	20.6	—	20.4	—
	Middle highest	18.4	—	18.3	—	19.8	—	20.6	—	17.5	—
	Highest	21.3	—	19.3	—	22.3	—	19.9	—	19.7	—
Urban residence		23.4	—	60.1	—	23.9	—	20.4	—	38.7	—
**Community norms (EA averages)**		n =	83	n =	100	n =	221	n =	110	n =	120
Percent of women who want to wait 2 or more years until next pregnancy		62.7	2.0	63.5	1.7	70.9	1.2	67.9	1.6	75.5	1.7
Percent of women having first births before age 18		24.6	1.5	14.0	0.6	26.6	1.2	28.4	1.1	19.6	1.1
Percent of women dissatisfied with family planning		17.7	1.5	19.1	1.5	9.9	0.8	17.7	1.2	13.6	1.2
**Demand Generation**											
Visited by health worker about family planning in last 12 months—individual		19.8	—	13.5	—	18.1	—	15.8	—	9.7	—
Percent of women who had heard family planning message in the last few months (EA average)		59.3	3.5	76.1	4.4	41.0	2.0	76.2	1.9	87.8	1.2

^1^ The associated unweighted Ns for these estimates are 778, 1075, 1956, 992, 2198 for Burkina Faso, Ghana, Ethiopia, Uganda, and Kenya, respectively.

### Ethical approval

Ethical approval for conducting PMA2020 was received from institutional review boards in each country (Burkina Faso- Comite D’Ethique Pour La Recherche en Sante, Ministere de la Recherche Scientifique et de L’Innovation, Ministere de la Sante; Ethiopia–Ethiopian Health and Nutrition Research Institute (EHNRI); Ghana—Kwame Nkruma University of Science and Technology (KNUST); Kenya- Kenyatta National Hospital–University of Nairobi Ethics Review Committee; Uganda—Makerere University School of Public Health Higher Degrees, Research and Ethics Committee and Johns Hopkins Bloomberg School of Public Health. All respondents were approached for informed consent before enrollment in the study. Participants provided verbal consent in Burkina Faso, Ethiopia, and Ghana in accordance with country specific approved consent procedures for low-literacy populations. Consent was recorded in a checkbox by the interviewer into the smartphone. Participants provided written consent in Kenya and Uganda. Married minors (15–17) were treated as adults in all countries. Unmarried minors (15–17) were treated as adults in Burkina Faso; in all other countries, consent was granted by the parent and assent given by the respondent (verbal consent/assent was given in Ethiopia and Ghana and written consent/assent in Kenya and Uganda). All consent procedures were approved by the relevant ethical review board.

### Variable measurement

#### Dependent variables

The dependent variable is a categorical variable with three options: 1) current use of a short-acting modern method of contraception (injectable, pill, emergency contraception, male or female condoms, and cycle beads) 2) current use of a long-acting modern method of contraception (female or male sterilization, implant, or IUD) or 3) non-use of a modern method of contraception.

In keeping with the conceptual framework, variables at the level of the individual, measuring personal background, household wealth, and exposure to program outreach factors, and at the community level, measuring SDP and normative factors, were included in the model. These are defined as follows.

#### Individual socio-demographic characteristics and household wealth

The woman’s age is measured in single years. Her education, defined as the highest level of school attended, is categorized as no schooling, primary, and secondary or higher. Parity, or the woman’s number of live births, is categorized as: 0, 1–2, 3–4 and 5 or more births, and residence is classified as urban or rural. Household wealth is measured using a factor score constructed from a principal components analysis of household assets and construction characteristics and divided into quintiles. In Burkina Faso and Niger, due to households being predominantly poor, there was minimal variation in wealth scores, thus their asset scores were categorized into tertiles.

#### Community norms

To capture community norms around fertility regulation, we constructed three measures based on the percentage of women who expressed the desire to limit childbearing by two or more years, who experienced early childbearing before age 18, and expressed dissatisfaction with family planning. All are calculated as cluster-level percentages within the EA, with the observation of the index woman removed from the calculation of the average. A woman was defined as being dissatisfied with family planning if she met one of two sets of conditions: 1) she discontinued use in the past 12 months and cited husband opposition, fear of side effects or health concerns, interference with the body’s natural process as the reason or 2) she wanted to delay a birth for two or more years but reported not using a method of contraception because of personal, social, partner, or religious opposition, fear of side effects, or concerns about health or interference with the body’s processes.

#### Exposure to demand generation efforts

Programmatic efforts to promote contraceptive use are considered demand-generating and are measured by one individual-level variable (whether the woman was visited by a health worker in the past 12 months who spoke to her about family planning), and one community-level variable (the percentage of women in the cluster who reported hearing or seeing family planning on the radio, television, or in magazines or newspapers in the past few months).

#### SDP factors

In this study we take advantage of geo-referenced data and included the distance to the nearest health facility offering contraceptive services from each household. Each woman’s household in the sample was matched to the nearest SDP that reported offering contraception and its characteristics were included in our analysis. Distance was calculated as the geodetic distance in kilometers between the two locations. Any Global Positioning System (GPS) points recorded for a household or SDP with an accuracy of greater than 10 meters were treated as missing. Fewer than 0.5% of observations in all datasets were treated as missing.

The other characteristics pertaining to service provision by the nearest SDP included the number of contraceptive methods for which counseling was provided, the number of long-acting methods provided, the number of short-acting methods provided, and whether a stock-out of any method of contraception was experienced at any time in the three months prior to the survey. A composite measure of the sector, type, and level of facility was defined with three categories: hospital (either public or private), lower-level public, and lower-level private.

### Analytic approach

We first conducted country specific univariate analyses of the selected individual, community, and demand-generation variables using the female samples. We then conducted country specific univariate analyses of the SDP variables using the overall SDP sample, not just the nearest facility linked to a sampled woman. We tested country specific bivariate linear regression analyses (not shown here), regressing modern contraceptive use on each independent variable of interest. To assess the structure and relative influences across covariates, we analyzed country specific multilevel multinomial logistic models including all independent variables, regardless of statistical significance in the bivariate results. The variables elected for inclusion in our multivariate analyses were guided by the conceptual framework to profile the different levels of influences on individual use of modern contraception, after assessing for correlation and variation across variables.

Our multilevel multinomial logistic model can be expressed as below.

Using nonusers as the base category, the probability *π_iks_* for woman i in area k using a short-acting methods is given by

$$\pi_{iks}=\mathrm{Pr}\left(c_{iks}=1|X\right)=\frac{exp(z_{iks})}{\sum_{ikn}exp(z_{ikn})}$$

The linear prediction for the short-acting method is

$$z_{iks}=X^{\prime }\gamma_{s}+u_{k}$$

and *γ_s_* is the associated vector of coefficients; *c_iks_* is a binary measure for woman *i* in area *k* denoting whether she uses a short-acting method (1) or not (0); *X* is the covariate matrix; and *u_k_* is the area-level random effects, which signal variation at the community level due to unobserved correlates.

Similarly, the formulas for the long-acting method are

$$\pi_{ikl}=\mathrm{Pr}\left(c_{ikl}=1|X\right)=\frac{exp(z_{ikl})}{\sum_{ikn}exp(z_{ikn})}$$

$$z_{ikl}=X^{\prime }\gamma_{l}+v_{k}$$

This model allows us to account for the hierarchical structure of the data where women are nested within communities.

An advantage of simultaneously modeling the multinomial variable (i.e. nonuse vs short-acting vs long-acting) over separate logistic models (i.e. nonuse vs short-acting; nonsue vs long-acting) is that the former allows the area-level random effects in the two contrasts to be correlated.

For both short- and long-acting methods, area-level random effects and error terms are assumed to be independent. We have applied survey weights to account for the differential selection probability of each woman. Specifically, variances have been calculated to account for clustering, strata, and design effects using the Taylor linearization method. Statistical significance is determined using 95% confidence intervals (CIs) and alpha values of 0.10, 0.05 and 0.01. All analyses were conducted using Stata Version 15.0 (StataCorp, 2017). The results in all tables are presented by individual country in order of increasing rate of modern contraceptive use.

To facilitate an interpretation of the coefficients, average marginal effects (AME) have been calculated and presented in the tables. In the discussion narrative they are shown in parentheses () along with the level of statistical significance. The AME denotes the average change in the probability of an outcome when a covariate increases by one unit.

## Results

Table 1 summarizes the sample characteristics for females across the five countries while Table 2 presents the characteristics of the SDP sample. The SDP characteristics are shown in two ways; first, those of the entire sample in each PMA round, and second, as the distribution by woman; that is, the percentage of women whose nearest health facility met each characteristic.

**Table 2:** Sample composition by variable type, country and survey year: Characteristics of total service delivery point sample and characteristics of nearest service delivery point to female sample.

		Burkina Faso		Ghana		Ethiopia		Uganda		Kenya	
Service delivery point		n =	132	n =	231	n =	444	n =	362	n =	338
Percent offering family planning (%)		92.4	—	93.5	—	97.5	—	93.4	—	97.3	—
Facility type (%)											
	Hospital	8.3	—	35.5	—	18.9	—	13.3	—	18.9	—
	Other public	75.0	—	33.3	—	67.8	—	53.6	—	61.5	—
	Other private	16.7	—	31.2	—	13.3	—	33.2	—	19.5	—
% with any stockout in the last 3 months		46.2	—	61.0	—	48.9	—	66.6	—	52.7	—
Mean number of methods counsel on		10.1	—	8.9	—	10.2	—	10.2	—	11.4	—
Mean number of short acting methods offered		4.7	—	4.3	—	3.6	—	3.5	—	4.5	—
Mean number of long acting methods offered		1.9	—	1.2	—	1.7	—	1.1	—	1.7	—
Nearest service delivery point—women		n =	3,114	n =	5,048	n =	7,328	n =	3,651	n =	4,831
Distance to nearest family planning SDP—individual (mean kms)		4.9	1.3	1.8	0.4	2.8	0.6	2.6	0.5	1.5	0.2
Facility type (%)											
	Hospital	1.0	—	18.9	—	2.3	—	5.6	—	10.5	—
	Other public	92.8	—	39.7	—	89.5	—	47.2	—	52.9	—
	Other private	6.2	—	41.4	—	8.3	—	47.2	—	36.6	—
% with any stockout in the last 3 months		46.5	—	60.3	—	56.0	—	73.6	—	55.8	—
Mean number of methods counsel on		10.7	—	9.8	—	9.6	—	9.8	—	10.1	—
Mean number of short acting methods offered		4.8	—	4.3	—	3.5	—	3.4	—	4.2	—
Mean number of long acting methods offered		1.8	—	1.1	—	1.3	—	0.8	—	1.4	—

There is significant variation across countries in the individual outcome of interest, modern contraceptive use, and the selected socio-economic and background characteristics. Modern contraceptive use among all women ranged from a low of 21.9% in Burkina Faso to a high of 45.9% in Kenya. Urban residence ranged from a high in Ghana, with 60.1% of the sample living in urban areas, to a low of 20.4% in Uganda. Despite being less urban than Ghana, women in Kenya had the lowest mean distance to the nearest SDP that offered family planning services (1.5 km). Burkina Faso had the least accessible services with the longest mean distance of 4.9 km between a woman’s household and the nearest SDP. However, women in Burkina Faso were more likely than other countries to receive a health worker visit in the past 12 months where family planning was discussed (19.8%), while women in Kenya were the least likely to be visited (9.7%). Short-acting contraceptive methods made up a smaller share of the method mix in Burkina Faso (51.5%) than in other countries.

The average percentage of women in each EA community who wished to delay or avoid getting pregnant for at least two years generally increased with the national mCPR. The percentages ranged from 62.7% in Niger to 75.5% in Kenya. Uganda had the highest community percentage of women who had given birth before age 18 (28.4%) and Ghana the lowest (14.0%). Relatively high percentages of women had been exposed to family planning in the last few months across different media, with Ethiopia the lowest at 41.0% and Kenya the highest at 87.8%. The country with the lowest community proportion of women reporting dissatisfaction with family planning was Ethiopia at 9.9%. The highest percentage dissatisfied was in Ghana at 19.1%.

The majority of health facilities in the sample in Burkina Faso, Ethiopia, Uganda, and Kenya were non-hospital public facilities, ranging from 75% in Burkina Faso to 53.6% in Uganda, while in Ghana there were approximately equal distributions of hospitals, lower level public, and private facilities (35.5%, 33.3%, and 31.2%, respectively). Recent stock-outs were reported by SDPs in all countries but were most common in Uganda, with two out of three facilities (66.6%) in the sample reporting a stock-out of at least one method in the past three months. The lowest reported stock-out experience was in Burkina Faso, where 46.2% of SDPs reported at least one stockout in the previous three months. The distributions are slightly different at the population level. The nearest facility for the majority of women was lower-level public facilities except in Ghana and Uganda; in Ghana and Uganda, for 41.4% and 47.2% of women, respectively, the nearest health facility was a lower-level private health facility. Overall, the patterns for the number of methods counseled, provided, and whether or not a stock-out was experienced did not vary largely when looking at the overall SDP sample compared to the nearest SDP sample when linked to female respondents.

Tables 3–5 show the average marginal effects from the country specific multi-level multinomial logistic regression models and include EA random effects. Table 3 compares short-acting users to non-users of modern contraception, Table 4 compares long-acting users to non-users of modern contraception, and Table 5 compares long-acting users to short-acting users. Tables 3 through 5 also show the average marginal effects of each model with all countries combined into a pooled analysis.

**Table 3:** Average marginal effects from the multilevel multinomial logistic regression model of individual women's probability of short-acting contraceptive use on community, demand generation, and service delivery point variables adjusted for individual level socioeconomic characteristics, by country.

		Pooled		Burkina Faso		Ghana		Ethiopia		Uganda		Kenya	
Model 1: Short-acting versus non-use (base outcome)													
**Community norms**													
Average percent of women who want to wait 2 or more years until next pregnancy		0.114	***	0.061		0.029		0.253	***	0.018		-0.110	
Average percent of women having first births before age 18		0.033		0.075		-0.229	*	0.132	*	0.051		-0.324	**
Average percent of women dissatisfied with family planning		-0.081		0.106	** **	-0.208	**	-0.032	** **	-0.198	**	-0.039	** **
**Demand generation**													
Visited by health worker about family planning in last 12 months		0.061	***	0.069	***	0.091	***	0.064	***	0.043	**	0.017	
Average percent who had heard family planning message in the last few months		0.049	**	0.078	*	0.048		0.056		0.090	*	0.188	*
**Service delivery point level**													
Distance to nearest family planning SDP(km)		-0.004	***	-0.002	** **	-0.002		-0.006	***	-0.001		0.004	
Facility type (reference = Hospital)													
	Other public	-0.006		0.105	***	-0.038		-0.002		-0.043		-0.018	
	Other private	0.034		0.107	***	-0.018		0.027		0.023		-0.003	
Any stockout in the last 3 months		0.003		-0.007		0.022		-0.017		0.004		0.021	
Mean number of methods counsel on		0.002		0.004		0.002		-0.001		0.001		0.001	
Mean number of short acting methods offered		-0.006		-0.020	***	-0.014	*	0.018		0.006		-0.012	
Mean number of long acting methods offered		0.008		0.009		0.003		0.013		0.014		0.002	
**Individual characteristics**													
Female age (reference = 15–19)													
	20–24	0.157	***	0.133	***	0.176	***	0.156	***	0.061	**	0.192	***
	25–29	0.150	***	0.118	***	0.147	***	0.136	***	0.043		0.233	***
	30–34	0.103	***	0.091	***	0.095	***	0.068	***	0.051		0.163	***
	35–39	0.066	***	0.031		0.062	***	0.027		0.049		0.113	***
	40–44	0.017		0.045		0.018		-0.022		-0.013		0.033	
	45–49	-0.070	***	-0.042	*	-0.037	**	-0.081	***	-0.120	***	-0.124	***
Female education (reference = None)													
	Primary	0.075	***	0.092	***	0.050	***	0.054	***	0.083	***	0.178	***
	Secondary or more	0.099	***	0.077	***	0.067	***	0.059	***	0.127	***	0.244	***
Parity (reference = 0)													
	1–2	0.151	***	0.046	***	0.024	*	0.214	***	0.134	***	0.303	***
	3–4	0.222	***	0.118	***	0.113	***	0.246	***	0.203	***	0.424	***
	5+	0.253	***	0.149	***	0.162	***	0.290	***	0.220	***	0.421	***
Household wealth (reference = Lowest)													
	Middle lowest	0.015		-0.022		0.023		0.006		0.006		0.040	
	Middle	0.033	***	-0.008		0.009		0.033	*	0.064	***	0.042	
	Middle highest	0.042	***	-0.008		-0.009		0.049	**	0.080	***	0.061	
	Highest	0.073	***	0.063	*	0.001		0.095	***	0.111	***	0.044	
Urban residence (reference = Rural)		-0.019		-0.080	***	0.008		0.009		0.018		-0.007	
Country (reference = Burkina)													
	Ethiopia	0.041	***										
	Ghana	0.018											
	Kenya	0.158	***										
	Uganda	0.000											
**Random effect**													
Model 1—EA level		0.483		0.210		0.300		0.672		0.130		0.421	
95% CI		0.396–0.589		0.089–0.496		0.181–0.494		0.483–0.935		0.129–0.130		0.259–0.684	
												

* p < .10

** p < .05

*** p < .01

**Table 4:** Average marginal effects from the multilevel multinomial logistic regression model of individual women's probability of long-acting contraceptive use on community, demand generation, and service delivery point variables adjusted for individual level socioeconomic characteristics, by country.

		Pooled		Burkina Faso		Ghana		Ethiopia		Uganda		Kenya	
Model 2: Long-acting versus non-use (base outcome)													
**Community norms**													
Average percent of women who want to wait 2 or more years until next pregnancy		0.092	***	0.057		0.062		0.129	***	0.011		0.048	
Average percent of women having first births before age 18		-0.047		-0.037		-0.132		-0.054		0.055		-0.104	
Average percent of women dissatisfied with family planning		0.000		0.131		-0.021		0.027		-0.091		-0.041	
**Demand generation**													
Visited by health worker about family planning in last 12 months		0.036	***	0.036	**	0.033	***	-0.003		0.022		0.050	**
Average percent who had heard family planning message in the last few months		0.033	*	0.030		0.045	*	0.048		0.006		0.057	
**Service delivery point level**													
Distance to nearest family planning SDP (km)		-0.003	***	-0.006	***	-0.002		-0.001		0.001		-0.015	**
Facility type (reference = Hospital)													
	Other public	0.019		0.105	***	0.031	*	0.025		-0.017		-0.034	
	Other private	0.040	**	0.005	**	0.036	*	0.005		0.019		0.006	
Any stockout in the last 3 months		0.003		-0.006		-0.011		-0.001		0.006		0.028	
Mean number of methods counsel on		0.000		-0.019	*	0.000		-0.002		-0.001		0.004	
Mean number of short acting methods offered		-0.004		0.032	*	-0.001		0.016		-0.005	*	0.002	
Mean number of long acting methods offered		0.012	**	-0.039	*	0.013		0.005		0.014		-0.002	
**Individual characteristics**													
Female age (reference = 15–19)													
	20–24	0.059	***	0.020		0.065	***	0.075	***	0.030		0.039	
	25–29	0.043	***	-0.049		0.022		0.068	***	0.022		0.065	
	30–34	0.030	**	-0.046		0.028	*	0.022		0.029		0.046	
	35–39	0.014		-0.066	*	0.018		-0.007		0.068	**	0.000	
	40–44	0.002		-0.091	**	0.001		-0.013		0.064	**	-0.012	
	45–49	-0.047	***	-0.122	***	-0.014		-0.061	***	0.005		-0.105	**
Female education (reference = None)													
	Primary	0.045	***	0.044	**	0.024	**	0.028	***	0.015		0.106	***
	Secondary or more	0.058	***	0.079	***	0.017	*	0.038	***	0.017		0.147	***
Parity (reference = 0)													
	1–2	0.116	***	0.140	***	0.034	***	0.130	***	0.062	***	0.238	***
	3–4	0.198	***	0.250	***	0.082	***	0.164	***	0.106	***	0.460	***
	5+	0.224	***	0.278	***	0.137	***	0.192	***	0.131	***	0.449	***
Household wealth (reference = Lowest)													
	Middle lowest	0.007		-0.015		0.020	*	-0.006		-0.010		0.031	
	Middle	0.020	**	-0.015		0.021	*	0.021		-0.004		0.064	***
	Middle highest	0.027	***	0.015		0.003		0.020		0.001		0.104	***
	Highest	0.039	***	0.041		0.004		0.018		0.036		0.107	***
Urban residence (reference = Rural)		0.002		-0.039		0.023		-0.005		-0.017		0.051	
Country (reference = Burkina)													
	Ethiopia	-0.035	**										
	Ghana	-0.079	***										
	Kenya	0.055	***										
	Uganda	-0.078	***										
**Random effect**													
Model 2—EA level		0.692		0.145		0.735		0.971		0.416		0.624	
95% CI		0.549–0.872		0.044-.480		0.393–1.376		0.662–1.424		0.415–0.416		0.364–1.070	
												

* p < .10

** p < .05

*** p < .01

**Table 5:** Average marginal effects from the multilevel multinomial logistic regression model of individual women's probability of long-acting contraceptive use relative to short-acting contraceptive use on community, demand generation, and service delivery point variables adjusted for individual level socioeconomic characteristics, by country.

		Pooled		Burkina Faso		Ghana		Ethiopia		Uganda		Kenya	
Model 3: Long-acting versus short-acting (base outcome)													
**Community norms**													
Average percent of women who want to wait 2 or more years until next pregnancy		0.006		0.086		0.084		-0.145		0.011		0.165	
Average percent of women having first births before age 18		-0.101		-0.185		-0.254		-0.232	*	0.047		0.168	
Average percent of women dissatisfied with family planning		-0.103		-0.196		0.054		-0.109		-0.013	*	-0.235	*
**Demand generation**													
Visited by health worker about family planning in last 12 months		0.001		-0.058		-0.007		-0.014		0.005		0.033	
Average percent who had heard family planning message in the last few months		-0.031		-0.154		0.087		0.012		-0.124		-0.132	
**Service delivery point level**													
Distance to nearest family planning SDP (km)		-0.001	***	-0.007	***	-0.006		0.003		0.004		-0.010	
Facility type (reference = Hospital)													
	Other public	0.035		0.125		0.135	**	0.024		-0.020		-0.022	
	Other private	0.011		0.079		0.143	**	-0.065		-0.039		-0.023	
Any stockout in the last 3 months		0.007		0.024		-0.063	*	0.024		0.007		0.007	
Mean number of methods counsel on		-0.002		-0.021	**	-0.002		-0.003		-0.002		0.002	
Mean number of short acting methods offered		-0.002		-0.007		0.021		0.016		-0.025	*	0.002	
Mean number of long acting methods offered		0.008	*	0.046		0.035	*	-0.014		0.012		-0.004	
**Individual characteristics**													
Female age (reference = 15–19)													
	20–24	-0.009		-0.135	***	0.040		0.039		0.027		-0.051	
	25–29	-0.029		-0.224	***	-0.041		0.032		0.019		-0.061	
	30–34	-0.010		-0.187	**	0.033		0.017		0.044		-0.039	
	35–39	0.009		-0.121		0.055		0.000		0.116		-0.048	
	40–44	0.049		-0.224	***	0.031		0.075		0.196	**	0.014	
	45–49	0.079	**	-0.070		0.104		-0.093		0.261	***	0.048	
Female education (reference = None)													
	Primary	-0.004		-0.054		0.045		-0.027		-0.080		-0.008	
	Secondary or more	-0.004		0.021		0.009		-0.018		-0.114	*	-0.004	
Parity (reference = 0)													
	1–2	0.190	***	0.403	***	0.124	***	0.123	***	0.193	***	0.262	***
	3–4	0.242	***	0.450	***	0.144	***	0.141	***	0.210	***	0.391	***
	5+	0.238	***	0.441	***	0.190	***	0.115	**	0.228	***	0.390	***
Household wealth (reference = Lowest)													
	Middle lowest	0.001		0.077		0.033		-0.037		-0.040		0.008	
	Middle	0.010		0.044		0.044		0.003		-0.096	**	0.040	
	Middle highest	0.017		0.067		0.004		-0.042		-0.080	*	0.084	***
	Highest	0.008		0.037		-0.018		-0.094		-0.059		0.108	***
Urban residence (reference = Rural)		0.036	*	0.094		0.018	*	-0.012		-0.077	*	0.029	
Country (reference = Burkina)													
	Ethiopia	-0.170	***										
	Ghana	-0.189	***										
	Kenya	-0.135	***										
	Uganda	-0.201	***										
**Random effect**													
Model 3—EA level		0.552		0.400		0.358		0.547		0.223		0.625	
95% CI		0.436–0.700		0.400–0.400		0.148–0.862		0.343–0.873		0.223–0.223		0.410–0.952	

* p < .10

** p < .05

*** p < .01

Based on the pooled data, if all women in a community wish to wait two or more years before their next birth, an average woman in that community would be 11.4 percentage points (p < .01) more likely to currently be using a short-acting contraceptive method and 9.2 percentage points (p < .01) more likely to be using a long-acting method than a woman in a community where all women wish to have a child within the next two years, holding other covariates at sample average levels (Tables 3 and 4). This community characteristic was positively associated with short-acting contraceptive use in four of the five country specific analyses but only reached statistical significance in Ethiopia (AME = 0.253, p < .01) (Table 3); the country specific results for long-acting method choice (Table 4) also shows statistical significance in Ethiopia (0.120, p < .01). Comparing long- versus short-acting contraceptive use, community-level desire to delay the next birth was not significantly associated with the likelihood of long-acting contraceptive use.

Community experience with early childbearing was generally not significantly associated with type of modern method used, while community dissatisfaction with family planning was associated with short-acting method use. The percent of non-users in the community who reported concerns or opposition to family planning was significantly and negatively associated with short-acting use in the pooled estimates in Ghana (-0.208, p < .05) and Uganda (β = -0.198, p < .05) (Table 3). Other things being equal, if all women in a community were dissatisfied with family planning, the predicted probability of short-acting contraceptive use would be 20.8 percentage points lower for an average woman in Ghana and 19.8 percentage points lower for a woman in Uganda compared to a woman in a community where no one expressed dissatisfaction with family planning (Table 3). This relationship did not hold for long-acting contraceptive use compared to non-use, or long-acting compared to short-acting contraceptive use (Tables 4 and 5).

With regard to demand generation, being visited by a health worker who discussed family planning in the last 12 months was consistently positively associated with short-acting contraceptive use across all countries, reaching statistical significance in the pooled results (0.061, p < .01) and in four country specific analyses: Burkina Faso (0.106), Ghana (0.091), Ethiopia (0.064), and Uganda (0.043) (Table 3). Similarly for long-acting contraception in the pooled analyses, all women in a community recently being visited by a health worker who discussed family planning was significantly associated with a 3.6 percentage point (p<0.01) increase in a woman’s likelihood of using long-acting contraception compared to women in a community where no women were recently visited by a health worker who discussed family planning (Table 4). This relationship was also significant in the country specific analysis for Burkina Faso (0.036, p<0.05), Ghana (0.033, p<0.01), and Kenya (0.050, p<0.05). Results comparing the likelihood of long-acting versus short-acting contraception revealed no significant association (Table 5). With regard to short-acting contraceptive use, greater exposure to family planning messages in the last few months among women in the community was associated with significant increases in a woman’s predicted probability of short-acting contraceptive use in the pooled analysis (0.049, p<0.05) and marginally significant increases in Burkina Faso (0.078, p < .10), Uganda (0.090, p<0.10) and Kenya (0.188, p < .10) (Table 3). Community-wide exposure to family planning messages was borderline related to the likelihood of long-acting contraceptive use overall (0.033, p < .10) and in Ghana (0.045, p < .10). It was not associated with a differential likelihood of short- versus long-acting contraceptive use.

Some aspects of the service delivery environment were significantly associated with contraceptive use in the pooled or country specific analyses. Distance to a health facility that offered family planning was associated with a lower probability of short-acting contraceptive use overall (-0.004, p < .01) and in four countries but statistically significant only in Ethiopia (-0.006, p<0.01). A one-kilometer increase in distance to the nearest SDP providing family planning for all women in a community was associated with a 0.4 percentage point decrease in a woman’s predicted probability of using short-acting contraception (Table 3). This relationship held in examining long-acting contraceptive use compared to non-use (-0.003, p < .01), but was only significant in country specific analyses in Burkina Faso (-0.006, p<0.01) and Kenya (-0.015, p<0.05). Distance’s negative effect was also observed with respect to use of a long-acting versus a short-acting contraceptive use in the pooled analysis (-0.001, p < .01) and for Burkina Faso (-0.007, p < .01). For the other characteristics of the nearest health facility offering family planning services, the relationships with use of a short-acting (Table 3) and long-acting (Table 4) versus no method were seen to be statistically significant most often in Burkina Faso and for short- versus long-acting method use in Ghana (Table 5). The type of facility appeared most often to be significantly associated with a woman’s use of short- or long-acting contraception. Positive AMEs were observed for other facility factors but only with occasional statistical significance. In particular, mean number of long-acting methods offered was significantly associated with long-acting versus no method use overall (0.012, p < .05) and only borderline significant in Burkina Faso, although negative (-0.039, p < .10) (Table 4), and for the comparison of a short- versus long-acting method overall (0.008, p < .10) and in Ghana (0.035, p < .10) (Table 5).

At the individual level, age was associated with short-acting contraceptive use in the majority of countries, although the relationship varied across countries (Table 3). Generally, women age 20–44 were significantly more likely to use short-acting contraception than women age 15–19, while women age 45–49 were significantly less likely to be using contraception in all countries and in the pooled estimates (-0.070, p < .01) (Table 3). Age was less associated with long-acting versus no contraceptive use (Table 4) but older age women favored long- versus short-acting methods (Table 5). Education was positively and significantly associated with contraceptive use across all three models in most countries (Tables 3, 4 and 5). In all countries, increased parity was significantly and positively associated with use of short-acting and long-acting contraceptive use (Tables 3, 4 and 5). Increased wealth was generally positively associated with short-acting contraceptive use overall and in Ethiopia and Uganda (Table 3), and long-acting contraceptive use overall and in Uganda and Kenya (Table 4), while the relationship was not statistically significant for the choice between a long- and short-acting method (Table 5).

The random effects parameter for the EA was statistically significant in all countries and in the pooled model for all analyses, indicating that there were significant unobserved EA characteristics related to modern method use not captured in our models. However, the variability in use levels due to EA random effects was minimal. Inclusion of the country-specific fixed effects in the pooled model show that, once adjusted for other factors, relative to Burkina Faso, women in Uganda, have similar individual probabilities of short-acting contraceptive use while those in Kenya have similar probability of long-acting contraceptive use. Conversely, relative to women in Burkina Faso, women in Ethiopia and Kenya had significantly higher individual probabilities of short-acting contraceptive use (0.041, p<0.01, 0.158 p<0.01, respectively) and women in Ethiopia, Ghana, and Uganda had lower individual probabilities of long-acting contraceptive use (-0.035 p < .05, -0.079, p < .01, and -0.078 p<0.01, respectively) (Tables 3 and 4). Women in all countries were also significantly less likely to be using long-acting compared to short-acting contraception than women in Burkina Faso (Table 5).

## Discussion

The aim of this analysis was to examine the multilevel relationship between community norms, demand generation efforts, factors in the contraceptive supply environment, and individual characteristics with the probability of short-acting and long-acting modern contraceptive use in five sub-Saharan African countries. By evaluating the effect of variables in each of these domains, we sought to show empirically where resource investments into health and development policy and program efforts may enable and raise the probability of contraceptive use.

The relationship with community-level normative behaviors was mixed. Living in an area where women expressed greater opposition to family planning was negatively associated with short-acting contraceptive use in every country except Burkina Faso, but it was only significant in Ghana and Uganda; there was no effect on long-acting method use. Desire to wait at least two years until one’s next birth was similarly significantly associated with greater short-acting and long-acting contraceptive use in the pooled analysis, but this relationship was driven by Ethiopia, the only country where these measures attained significance. Early childbearing was not consistently related to short-acting contraceptive use. That most countries showed a consistent pattern of negative association between modern contraceptive use and the proportion of women having unfavorable opinions about contraception suggests that community perceptions can exert influence on women’s choice to use family planning, a finding in keeping with other studies [6,15], but the effect may not be as large as expected. Additionally, some studies have posited a relationship between cultural norms and method mix [16,17]. Our study found evidence that normative influences in the community may negatively affect the use of a short-acting method, but we found little evidence that normative influences affect use of a long-acting method. The influence of community norms on method choice when comparing short- and long-acting users is also less evident, suggesting these may play a larger role with respect to using any method as opposed to the type of method.

One measure of demand generation, being visited by a health worker, was significantly and positively associated with short-acting contraceptive use in Burkina Faso, Ghana, Ethiopia, and Uganda and positively associated, though not significantly, in Kenya. This relationship was significant and positively associated with long-acting contraceptive use in Burkina Faso, Ghana, and Kenya. A second measure of demand generation—exposure to family planning messages in the media at the community level—did not have the expected relationship in the multivariate model, only reaching modest significance (p < .10) in Burkina Faso, Uganda, and Kenya with regard to short-acting contraceptive use and Ghana with regard to long-acting method use. This finding is consistent with Skiles and colleagues’ work in Malawi, which found increased use of injectables among women visited by a health worker, but no separate association between contraceptive use and exposure to mass media campaigns [18]. However, the lack of association in the multilevel models of exposure to multimedia campaigns and contraceptive use is in contrast to a recent study that showed a positive relationship [19]. Neither media exposure nor health worker visit showed a statistically significant relationship with the choice of a long- versus short-acting method in our study. This suggests that health workers are a critical source of information, introducing women to contraceptives in general, a finding that has been well-documented [20–22], but may have less influence on method choice. The form of community-based distribution—that is, what methods community workers are allowed to provide—varies widely across countries, but the consistency of the association between receiving a home visit and using a method underscores the importance of these outreach efforts in increasing demand for methods.

The relationship of distance to the nearest health facility and contraceptive use was largely negative, as expected, although not generally statistically significant across the five countries. Previous studies have found varying results for the association of distance to services on contraceptive use [19,23–27]. Pooled and country specific results for Ethiopia showed distance to be significantly and negatively related to short-acting contraceptive use, while only Burkina Faso and Kenya results demonstrated a significant negative association for long-acting contraceptive use compared to none. Only Burkina demonstrated that distance negatively impacted the probability of using a long-acting method relative to a short-acting method, among users. Given the positive and significant associations from recent health worker visits in Burkina Faso, Ghana, Ethiopia, and Uganda, this outreach may mitigate the effects of distance on short-acting contraceptive use. Conversely, distance was significantly negatively associated with long-acting contraceptive use in Burkina Faso and Kenya, as was being visited by a health worker recently. These findings support the idea that distance to a health facility is a greater barrier for methods that require a trained provider, but that fieldworker outreach can, at least partially, alleviate this obstacle.

Distance aside, characteristics of the nearest health facility did not exercise their own independent influences on a woman’s use of a short-acting method, which is consistent with other research [28]. Characteristics of the service environment gained modestly greater significance when a user’s choice of a long-acting methods versus short-acting methods or non-use was examined, but the majority of associations were weak. The exception to these patterns is Burkina Faso, where characteristics of the service environment six-months prior to the individual survey demonstrated significant impact on use of short-acting and long-acting method use six months later. Distance to the nearest health facility that offers family planning is almost twice as high in Burkina Faso as other countries and the greater effect of the service delivery environment on use likely reflects that women in Burkina Faso have fewer options for service providers. When a woman has access to a greater range of providers, the individual characteristics of one provider may not affect use, as she can find alternative providers; in Burkina Faso, however, where access to a provider is limited, the individual characteristics of that single provider may significantly impact her ability to obtain and use a method of contraception.

Although the effect size of the community-level random intercepts was small in all countries (0.13–0.75), they were consistently significant in the multilevel model. This indicates that there is significant variation between communities that is not accounted for by our observed measures. Although we were able to include a variety of variables that measure different components of demand generation, norms, and the service delivery environment, additional cultural, religious, and contextual factors likely play important roles in contraceptive use decision-making. Inclusion of country fixed effects in the pooled models support the conclusion that there is significant variation across countries in how community, individual, and service delivery environment factors influence contraceptive use.

Our study has several limitations. First, PMA2020 does not field questions on community norms, thereby requiring us to estimate these via community level averages of individual responses. This is a common practice in the literature [6,15,29], but questions specific to normative influences and appropriately built attitudinal scales would strengthen our understanding of how enduring these factors’ influences are. For example, the measurement of concerns or opposition regarding family planning was based on responses from women not currently using a contraceptive method. Women using a method are assumed to be satisfied, as is the approach taken with defining met need and satisfied demand [30]. However, it is possible that users may contribute to the perpetration of myths and misconceptions based on their contraceptive experiences. This restricted measurement approach can lead to an over-estimation of the effect of community dissatisfaction with contraception on individual use.

A second limitation is that we attributed influence from the service characteristics of the nearest SDP to an individual woman’s contraceptive use, although that may not be where she seeks care or obtained her method. We defined service characteristics of the nearest SDP to represent an optimal ecology of contraceptive access for women; this definition relies on two assumptions: 1) that women prefer facilities based on distance and 2) that geodetic distance is a reasonable proxy for access. In terms of the first assumption, in countries such as Burkina Faso, Ethiopia and Niger, where the average distance to a health facility is over 5 km and service density is low, this likely is a reasonable assumption. We recognize, however, that the decision of where to obtain services, particularly in countries with a high density of source choice, is likely driven by more than just distance. Women may choose to go to facilities that are farther away for reasons such as confidentiality, travel convenience, wait time, or quality [31–33]. Optimally, we would be able to match women with the facilities from which they obtain services, however, obtaining accurate reports from women of the names and locations of facilities where they obtained contraception is challenging and we do not have this information. Additionally, in order to compare the effect of the service delivery environment on use or non-use of a modern contraceptive method, it is necessary to link non-users with some facility as a reference. As they have not, by definition, obtained their method from a specific facility, we chose to use the nearest facility as that reference. In terms of the second assumption, there are multiple ways to conceptualize and operationalize access, including, but not limited to, service availability, financial availability, and social and cultural access [34,35]. Much of research and program efforts, however, focus on improving service availability [18,36,37], which thus guided our approach to defining the available constellation of family planning provision among sampled women by distance and by health worker outreach. Other researchers have defined the family planning service delivery environment differently, for example by examining the average characteristics of numerous facilities within a certain range of an individual female [18,28] or by using road distance or travel time as alternative measures of distance [18,38]. Variation in how these service delivery environment variables are constructed may have implications for the associated findings.

At the same time, this study has a number of strengths. First, we were able to incorporate distance measured at the household, rather than cluster level, which increases variation and accuracy. Additionally, we used data from health facility and household surveys that were collected six months apart so that we could build in a lag for influence of the health service environment on individual behavior, using health facility factors present six months prior to the measured contraceptive behavioral outcome. Third, by using multilevel models, we were able to account for clustering at the community level and examine the extent to which the choice to use short- or long-acting contraception, and the choice to use a short- versus long-acting method, vary by community. Finally, we conducted this analysis using nationally representative data from five sub-Saharan African countries that are important either for their population size or Francophone West Africa regional location. This allowed us to investigate and observe patterns in these relationships across programmatic and development contexts. While the pattern of associations was variable across countries, there were also apparent consistencies in the influence of service provision, access, and personal background factors that reinforce this study’s ability to make comparisons and draw conclusions.

## Conclusion

Use of modern contraception in the five sub-Saharan African study countries has been trending upward in recent years, suggesting that demand is increasingly satisfied either through individual motivation (Bongaarts 2017) or facilitated by improved systems of contraceptive delivery. This analysis, enhanced by multilevel modeling of linked household, female, and SDP data, shows that access and service provision characteristics of proximal health facilities can influence contraceptive use decisions when choice of provider is limited, independent of community norms and individual factors. Additionally, dissatisfaction with contraceptive use at the community level can significantly affect use patterns and should be considered when designing policies and programs. Investments to decrease distance to services, improve satisfaction with methods, and increase health worker outreach are likely to increase modern method use.
